# Fine-Tuning of Gene Expression by tRNA-Derived Fragments during Abiotic Stress Signal Transduction

**DOI:** 10.3390/ijms19020518

**Published:** 2018-02-08

**Authors:** Eun Joo Park, Tae-Houn Kim

**Affiliations:** Department of Prepharm-Med/Health Functional Biomaterials, Duksung Women’s University, Seoul 01369, Korea; cokun2013@duksung.ac.kr

**Keywords:** tRNA derived fragment, abscisic acid, abiotic stress, post-transcriptional gene silencing, plant

## Abstract

When plants are subjected to unfavorable environmental conditions, overall gene expression in stressed cells is altered from a programmed pattern for normal development to an adaptive pattern for survival. Rapid changes in plant gene expression include production of stress responsive proteins for protection as well as reduction of irrelevant proteins to minimize energy consumption during growth. In addition to the many established mechanisms known to modulate gene expression in eukaryotes, a novel strategy involving tRNA-derived fragments (tRFs) was recently reported to control gene expression. In animals, tRFs are shown to play a certain role in infected or cancer cells. However, tRFs are expected to function in the regulation of gene expression against abiotic stress conditions in plants. Moreover, the underlying mechanism linking up-regulation of tRFs under stress conditions with the stress tolerant response remains unknown. In this review, the biogenesis and putative function of diverse tRFs in abiotic stress signaling are discussed with a focus on tRFs as a transcriptional/post-transcriptional/translational regulator.

## 1. Introduction

In response to a stressful environment, plants must coordinate intricate signaling pathways that lead to wide physiological changes to enhance viability under harsh conditions. The plant hormone abscisic acid (ABA) acts as an essential molecule to initiate various tolerant response mechanisms against unfavorable conditions [1,2,3]. Much of the regulation of ABA signaling and the stress response depend on the control of gene expression [4]. One such candidate that participates in the regulation of ABA-dependent or stress-dependent gene expression is miRNAs. Prior reports have demonstrated that miRNAs may function in the control of gene expression under abiotic stress conditions [5,6]. Recently, small RNAs derived from tRNAs were reported to be enriched in cells under stress conditions, and these are considered as novel functioning ncRNAs during stress responses.

**Figure 1:**
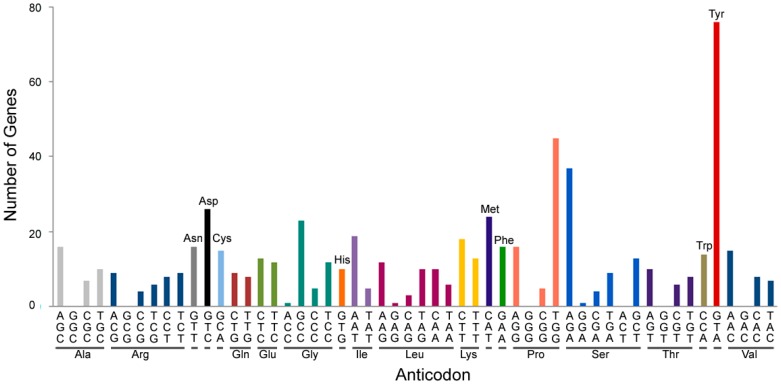
Multiple tRNA genes in *Arabidopsis*. Numbers of tRNA genes are presented for different amino acids and anticodons. Each graph with different colors indicates a different amino acid. Various isoacceptors (tRNA acceptors that accept the same amino acids) exist for each amino acid. Existence of isodecoders (tRNA genes with the same anticodon but different sequences elsewhere in the tRNA body) for each isoacceptor expand the source of tRNAs for generation of diverse tRFs.

As seen in many other organisms, tRNAs are encoded by multiple genes in *Arabidopsis* (Figure 1). Since there are many isoacceptors (tRNA acceptors that take the same amino acids) for each amino acid and isodecoders (tRNA genes with the same anticodon but different sequences elsewhere in the tRNA body) for each isoacceptor, various types of tRNA fragments (tRFs) can be generated from diverse tRNA sources. Moreover multiple genes for tRNA modification as shown in *Arabidopsis* [7] increase the complexity of tRF biogenesis in a cell type-specific or stress-specific manner. In light of the importance of this new subject, there have been several reviews on the biogenesis and putative function of tRFs focusing on cancer and pathogen-associated stress conditions [8,9,10,11]. In this review, we present recent findings on tRF biogenesis and discuss their putative function especially under abiotic stress conditions.

## 2. tRNA-Derived Fragments Are Small ncRNAs with a Specific Function

**Figure 2:**
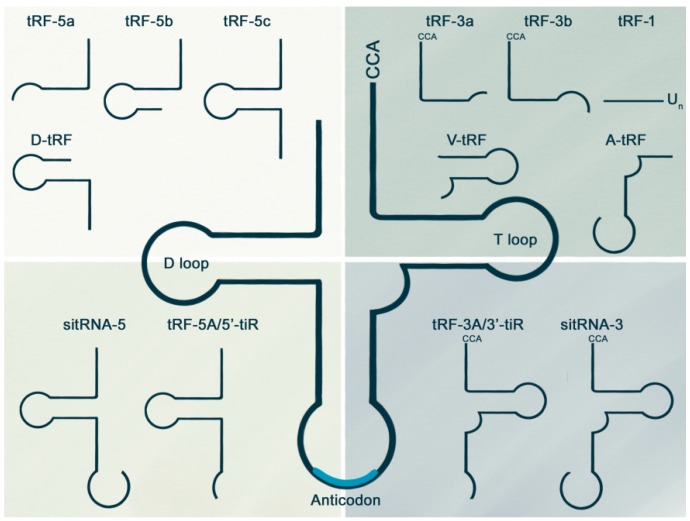
Several types of tRFs are classified by size and sequence location in the tRNA structure. tRFs are generated during pre-tRNA processing or from mature tRNAs. tRF-1 is generated by RNaseZ, which cleaves 3′ trailer sequences from pre-tRNA. Half-sized tRFs are grouped by whether their source sequences are from 5′ or 3′ fragments. Longer tRFs than tRNA halves are termed sitRNAs. In the case of short-length tRFs, subtypes are determined by size and location of the source. See the following references for detailed classification [8,11,18,23,26,28].

In eukaryotes, generation of mature tRNAs begins with the transcription of pre-tRNA, consisting of a 5′ leader sequence and a 3′ trailer sequence, by RNA polymerase III. The 5′ and 3′ extra sequences are then excised, and a CCA terminal nucleotide is added to the 3′ end of the tRNA intermediate through the multistep enzymatic process of tRNA maturation. In most cases, tRFs are generated through two different pathways, either as byproducts of pre-tRNA processing or from mature tRNAs. Each pathway generates different types of tRFs depending on whether the processing target site is located in the D or T arm with various subtypes (Figure 2). tRFs with amino acid specificity have been detected in stress-specific manners in yeast, mouse, and *Arabidopsis* [12,13,14,15,16,17]; in tissue-specific manners in mouse, human, and rice [18,19,20]; and in developmental stage-specific manners in the fungus, *Aspergillus fumigatus* [21]. However, overall end sequence conservation among all reported tRFs across various species strongly suggests that biogenesis of tRFs is under the control of a precise mechanism and might be involved in specific biological processes.

As more than 90% of RNA modifications occur on tRNA (The RNA Modification Database: http://mods.rna.albany.edu/mods), multiple enzymes governing tRNA modification have been identified in cells [7,10,22]. The tRNA quality control process begins during pre-tRNA processing and continues until after processing. Misprocessed pre-tRNAs are subjected to a nuclear surveillance pathway, and hypomodified or misfolded tRNAs after processing are targeted for rapid tRNA decay [8]. In comparison to the inconsistent degradants produced by 5′ to 3′ or 3′ to 5′ exonucleases in the classical degradation pathway, tRFs of consistent length are generated from a specific portion of the tRNA structure. As illustrated in Figure 2, tRFs can be generated during pre-tRNA processing or from mature tRNAs. tRFs can be grouped depending on the tRNA part used in the synthesis of tRFs [8,11]. Mature tRNAs provide versatile sources for generation of numerous tRF subtypes. The tRNA halves formed by cleavage at the anticodon loop are named as tRF-5A/5′-tiR for the 5′ segment of tRNA and tRF-3A/3′-tiR for the 3′ segment of tRNA [8,11,23]. There have been numerous reports that these tRNA halves increase in number in response to various types of stress conditions such as oxidative stress in *Arabidopsis*, yeast, and *Drosophila* [12,13,14,24]; nutrient-deficient conditions in *Tetrahymena* and *Aspergillus* [21,25]; and hypertonic stress conditions in mouse embryonic fibroblasts [15]. Longer fragments than tRF-3A have been detected in *Giardia lamblia* [26] and were designated as sitRNA-3 (stress induced tRNA-3) for the 3′ segment of tRNA and sitRNA-5 for the 5′ segment of tRNA. Short-length fragments are much more diverse than half-sized ones with diverse cleavage sites within the tRNA structure. The 5′ region covering the D-arm can be termed tRF-5 with subtype a/b/c [11]. tRF-5a and tRF-5b are formed by cleavage at the D-loop and D-stem, respectively. tRF-5c is produced by cleavage at the anticodon stem. tRF-3a and tRF-3b are generated by cleavage at the T-loop. Similar to tRNA halves, generation of these short-length tRFs responds to various stress conditions. For instance, tRF-5a was detected in *Arabidopsis* root under phosphate-deprived conditions [16], oxidative stress, and drought stress [12]. tRF-5c was observed in low protein-fed mouse sperm [17], and recently this fragment was reported to be generated from tRF-5A [27]. tRFs produced from the internal region of tRNA are named based on where the 5′ end starts in the tRNA. Especially, D-tRF indicates fragments formed after cleavage in the D-stem. A-tRF and V-tRF are formed from cleavage at the anticodon loop and variable region, respectively. These variants were specifically detected in prostate cancer cells, indicating their specific roles in excessive cell proliferation [28]. Similarly, an increased level of tRF-1 was produced in cancer cell lines by RNaseZ during pre-tRNA processing in response to nutrient-deficient conditions [18].

It is intriguing that certain tRFs generated from tRNAs for specific amino acids were shown to be specifically produced upon a particular stress stimulus or in a tissue-specific manner. These stress-specific or developmental stage-specific tRFs from tRNAs for different amino acids are believed to play distinct roles in corresponding cellular responses. In wheat seedlings, tRFs-3a/b derived from tRNA^Val-CAC^, tRNA^Thr-UGU^, tRNA^Tyr-GUA^, and tRNA^Ser-UGA^ were found to be highly induced under salt stress [29]. In silico analysis of *Arabidopsis* RNA sequencing libraries revealed that tRF^Ala-AGC^-5a was induced under salt stress, and tRF^Arg-TCG/Gly-TCC^-5a was highly elevated by drought stress [30]. Furthermore, tRF^Asp^-5a was found to be weakly expressed compared to tRF^Gly^-5a under phosphate-deficient conditions [16].

A tissue-specific enrichment pattern for tRF-5/3 was observed in *Arabidopsis* [12]. For example, tRF^Glu-CTC^-5A was detected in flowers but not seedlings [13], and tRF^Asp^-5a induced by phosphate conditions was only detected in roots but not in shoots [16]. Alteration of tRF levels in undifferentiated and differentiating calli from rice [19] further implies the developmental role of tRFs possibly involving epigenetic regulation in the nucleus. This tissue-specific tRF enrichment might be a general signaling event since similar results have been reported in many other species [18,20]. A developmental stage-specific function of tRFs was observed in *Phytophthora infestans* [31], *Aspergillus* [21], and *Phytophthora sojae* [32]. Based on these observations, it is suggested that diverse tRF types exist in organisms possibly with distinct functions during stress responses or tissue-specific developmental processes. Future research including ectopic/tissue specific expressions of the particular tRFs or tissue specific small RNA profiling will answer to this hypothesis.

## 3. Biogenesis of tRFs Possibly Occurs through Dicer-Dependent and -Independent Pathways

**Figure 3:**
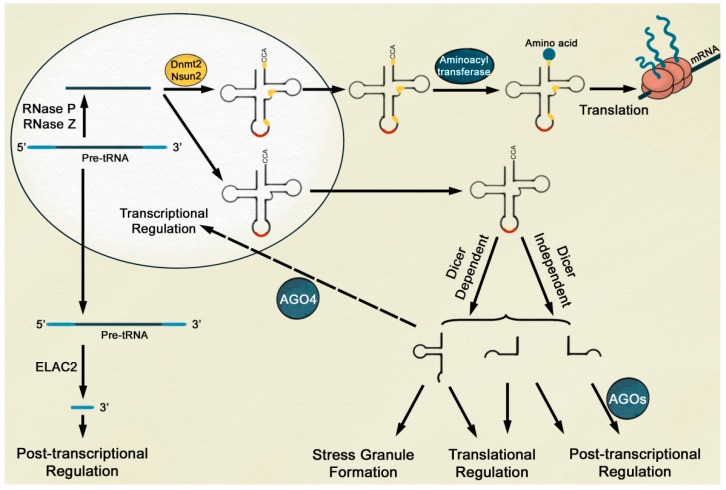
A proposed model on the diverse function of tRFs in gene expression during stress responses. Whereas mature tRNAs methylated by Dnmt2 (DNA methyltransferase 2) and Nsun2 (NOP2/Sun RNA methyltransferase family member 2) (yellow mark) participate in translation, unmethylated mature tRNAs or pre-tRNAs are processed into various types of tRFs [9,11,24,43]. Production of tRFs is promoted by abiotic stress conditions to eventually control gene expression by transcriptional, post-transcriptional, and translational regulation.

Considering tRFs can be generated from either pre-tRNAs or tRNAs, different processing components are likely to be involved in the biogenesis of tRFs depending on the starting material. Firstly, mature tRNAs might be subjected to the miRNA processing pathway involving Drosha and Dicer in the nucleus and cytoplasm, respectively, since processed tRF is similar in size and shows comparable subcellular localization with miRNA (Figure 3). In line with this idea, 19—22 nt tRF-5a and tRF-1 have been reported to be localized to the cytoplasm by several groups [18,33,34,35,36], and the level of tRF-5a/3b was reduced in *dcr* (*dicer*) knockdown mutants of human cell lines or *Arabidopsis* [33,34,35,37]. Furthermore, overexpression of recombinant Dicer1 in human cells has been shown to cause accumulation of tRFs, indicating that generation of tRFs and miRNAs shares the same processing apparatus [33]. Secondly, evidence for tRF formation through a Dicer-independent pathway was investigated in *Arabidopsis* [12] and many other eukaryotes [31,38,39]. This hypothesis was based on prior findings that some *dcr* mutants actually produce increased levels of tRFs [12,38,39]. tRFs are generated by Dicer and related processors under normal conditions. However, when the Dcr-dependent process is blocked, in this case by a mutation in Dcr, a Dcr-independent pathway might be employed to activate an alternative biosynthesis pathway for tRF production. However, further experimental tests are required to determine whether there is a switching mechanism for governing a selection between those two different pathways.

In the case of the Dicer independent pathway, different kinds of tRNA processing components are required for tRF biogenesis instead of Dicer. For example, reports have shown that RNase Z processes 3′ pre-tRNA into tRF-1 while 5′ tRNA processing enzyme converts the 5′ leader sequence into tRFs [18,28,34]. tRFs generated from mature tRNAs are fragmented by Angiogenin (ANG), which belongs to the RNase A family. The observation that heterologous expression of ANG in *Xenopus* oocytes produced tRNA-specific cleavage activity as well as inhibited protein synthesis suggests that ANG is a critical component of the Dicer-independent pathway [40]. In addition, Yeast Rny1p, a member of the RNase T2 family that translocates from vacuoles into the cytosol in response to oxidative stress, is known to produce tRNA halves and inhibit cell growth [14]. Likewise, the *Arabidopsis* RNase T2 family gene *RNS1* (*Ribonuclease 1*) was shown to be involved in the formation of tRF-5a/3b [12].

Considering diverse tRFs with different lengths and sequences, additional steps after excision of the internal site and other regulatory components besides ANG are necessary for generation of specific tRFs under certain conditions. For instance, it was reported that oxidative stress-induced tRNA halves highly accumulated when the ANG inhibitor RNH1 (Ribonuclease/angiogenin inhibitor 1) was down-regulated in mice [15] and humans [23]. Another crucial step for specific biogenesis of tRFs is tRNA modification. For example, *Drosophila* Dnmt2 (DNA methyltransferase 2) protects tRNA from cleavage via methylation. Mutant Dnmt2, which lacks methylation at the C38 of tRNA, was shown to be more responsive to oxidative stress and accumulated more tRNA halves under heat stress [24]. It was also reported that Dnmt2 translocated from the nucleus to stress granules (SGs) in response to oxidative stress treatment [41]. In addition, Nsun2/TRM4 (NOP2/Sun RNA methyltransferase family member 2/tRNA-(m5C) methyltransferase), another cytosine-5 tRNA methyltransferase, was shown to methylate different sites from the target area of Dnmt2 [42,43]. In the mouse knockout mutant Nsun2, unmethylated tRNAs showing enhanced interactions with ANG resulted in accumulation of tRF-5A [43]. Similarly, the knockdown mutant Nsun2 in the silkworm, *Bombyx mori* showed an increased level of tRF-5A/tRF-5c (td-piRNA) [27]. In *Arabidopsis*, different methylation patterns of tRNA were observed in response to the actions of TRDMT1 (tRNA aspartic acid methyltransferase 1)/Dnmt2 and TRM4B (paralog of Nsun2/TRM4) [44]. Based on this result, it is predicted that diverse tRNA modification enzymes differentially mark tRNAs according to the amino acid type [7,44], which eventually leads to the generation of specific tRFs (Figure 3). Furthermore, specific subcellular localization of certain types of tRFs can contribute to the production of variable tRF patterns, as shown by the preferential localization of tRF-1/tRF-3 in the cytoplasm and tRF-5 in the nucleus [38].

## 4. tRFs Are Involved in Post-Transcriptional and Translational Gene Silencing

A novel function of tRFs in the down-regulation of target genes was predicted based on their interactions with well-known RNA silencing components known as AGOs (Argonautes). Interactions between tRFs and AGOs occur in a specific combination, implying that a certain type of tRFs is preferentially recognized by a specific AGO. For example, tRF-5a was shown to interact with AGO1/2 [33,35] while tRF-3b was shown to interact with AGO2 [37,39]. Moreover, *in silico* analysis of AGO-Immunoprecipitation (IP) libraries in *Arabidopsis* revealed that tRF-5 dominantly existed in several AGO-IP fractions except AGO2-IP, which showed a stronger interaction with tRF-3b [30]. Considering that only AGO2 contains a slicer domain among AGOs in mammals [45], the AGO2-tRF-3b interaction might cause repression of the antisense reporter by directly cleaving target transcripts. Interestingly, specificity of tRF-AGO interactions is determined based on tRFs from tRNAs for a certain amino acid type. Immunoprecipitation of AGO2 revealed a stronger interaction of AGO2 with tRF^Leu-CAG^-3b than with tRF^His-GTG^-3b [39]. Similar results were observed in rice, as AGO1 showed an enhanced interaction with tRF^Arg-CCT^-5a but not with tRF^Ala-AGC^-5a [12]. Furthermore, interactions between tRFs and AGOs could be enhanced under specific stress conditions. Co-immunoprecipitation analysis of AGO1 demonstrated increased affinities to tRF^Gly-TCC^-5a under UV conditions [36], indicating that the different affinities of tRFs toward AGOs might be regulated by stress conditions.

Putative targets of tRFs could be predicted based on the tRF sequences, as shown in the case of miRNA-AGO interactions. For instance, transposable elements or long terminal repeats containing a matched sequence to tRFs have been discovered as putative tRF targets in mouse, rat, human, and *Arabidopsis* [17,35,38,39,46,47]. However, precise prediction of target sequences for tRFs requires more investigation since non-canonical seed sites of miRNAs were often identified depending on the neighboring sequence context of target sites [48,49].

Interestingly, some tRFs were shown to interact with AGO4. Since AGO4 participates in RNA-directed DNA methylation, tRFs interacting with AGO4 are expected to function beyond post-transcriptional gene silencing [50]. tRF-5a and 5A with sizes ranging from 18 to 27 nt were identified as AGO4 interacting partners by analyzing publicly available deep sequencing data for *Arabidopsis* [12,30]. tRF-3b was shown to interact more strongly with AGO3/4 than with AGO1/2 [34]. Moreover, *Tetrahymena* Piwi protein Twi12 was also found to interact with tRF-3b, which then translocated into the nucleus. In coordination with Xrn2 and Tan1, the tRF-3b-Twi12 complex regulates rRNA processing [51,52,53]. Given that AGO4 mounted with siRNA translocates into the nucleus [50,54], cytoplasm-localized tRFs bound to AGO4 may translocate into the nucleus and function in transcriptional or post-transcriptional gene silencing.

tRNA modification is closely related with increasing translational fidelity. For example, TRM4 methylates cytosine in tRNA at several locations and especially the modified m5C at the wobble position, resulting in enhanced translation of oxidative stress responsive genes containing specific anticodons [55]. In this context, *Arabidopsis* mutants for the *TRDMT1*/*Dnmt2* and *TRM4B* genes showed increased sensitivity to antibiotics, indicating defects in translation [44]. In addition to translational regulation by tRNA modification, tRFs produced after tRNA modification provide additional control for translational regulation by interfering with the binding of ribosomes to mRNA or formation of the translation initiation complex. For example, tRF^Val^-5b was identified as a ribosome-associated RNA under salt and pH stress conditions in the halophilic archaea *Haloferax volcanii*, and induction of tRF^Val^-5b was shown to reduce translation. This phenotype was complemented by mutation of tRFs, further supporting the hypothesis that tRFs bound to ribosomes might inhibit efficient translation by competing with mRNA for ribosome-binding sites [56,57]. Moreover, the inhibitory effect of tRFs on translation by targeting formation of the eIF4F complex in human cell lines suggests a regulatory function for tRFs in the control of gene expression [58,59].

## 5. Function of tRFs during Abiotic Stress Signal Transduction in Plants

Although biogenesis and the exact function of tRFs have not been revealed in plants, several reports have identified specific tRFs in response to certain stress conditions as well as their possible roles in stress tolerant cellular responses. tRF-Gly-TCC was shown to be elevated under UV treatment and detected in the fraction of AGO1 immunoprecipitation [36]. Similarly, an increase of tRF-Arg-5a and the interaction of this tRF with AGO1 was observed in drought condition [12]. Until now, plant genes found to be involved in the generation of tRFs under abiotic stress conditions are DCL1 (Dicer-like 1) and RNS1. Identification of the DCL1-dependent pathway was confirmed based on reduction of tRF^Ala^-5a in pollen of the *dcl1-11* mutant [35]. One of the factors involved in the Dicer-independent pathway, RNS1, was shown to be induced under ABA treatment, and the amount of tRFs increased in the RNS1 overexpression mutant [12,60]. Based on this finding, it is hypothesized that generation of tRFs can be induced by ABA via an RNS1-dependent pathway. In addition, lack of tRNA methylation in the *trdmt1* and *trm4* mutants [44] suggests that TRDMT1/Dnmt2 and TRM4/Nsun2 in plants are involved in tRF formation. Moreover, various tRNA modification enzymes present in *Arabidopsis* have the potential to participate in the biosynthesis of tRFs [7]. In line with this idea, a recent study has reported that the level of tRNA modification was indeed altered in the salt stress treatment [61]. Considering abiotic stress-induced tRF-AGO interaction was reported in the aforementioned studies, novel tRNA modifications other than conventional enzymes such as Dnmt2 and Nsun2 might be involved in tRF formation in plants depending on stress conditions.

A recent study on the inhibition of transposable elements identified AGO1-tRF as a critical component for silencing transposable elements [35]. In this research, tRF-5a observed in pollen was confirmed to interact with AGO1 in a series of pull-down experiments, and it inhibited expression of the transposable element *Athila6A* post-transcriptionally in a DCL1-dependent manner. Considering that Dnmt2 plays an important role in the formation of tRF, interaction of Dnmt2 with histone deacetylase HD2C (Histone Deacetylase 2C) in the nucleus suggests that tRFs represses gene expression by affecting histone modification, at least in *Arabidopsis* [62]. In fact, reduced ABA sensitivity was observed in HD2C overexpression lines while a hypersensitive ABA phenotype was observed in the *hd2c* mutant. These altered ABA phenotypes were accompanied by both reduced and enhanced ABA-responsive gene expression [63,64]. Close correlations between increased levels of specific tRFs and abiotic stress signal transduction suggest that tRFs could function in reduction of stress-related gene expression via transcriptional, post-transcriptional, and translational regulatory pathways. By switching from functional tRNAs for enhancing translation into inhibitory tRFs for suppression of normal growth, tRFs might provide a sequence-specific tool for the fine tuning of gene expression in response to stress conditions.

## 6. Conclusions

A novel function of tRNA beyond its well-known role as translational machinery has been recognized in diverse species. With the advent of deep sequencing technology, novel small non-coding RNAs derived from tRNAs were identified as tRNA-derived fragments (tRFs). The observation of altered tRF levels followed by stress conditions such as oxidative, nutrient deficiency, or heat stresses suggests that tRFs play a specific role during stress tolerant responses [12,13,14,21,23,24,25,65]. tRFs with lengths ranging from short to long could be generated from the D or T arm of pre- or mature tRNAs in Dicer-dependent and -independent pathways with the aid of RNase A/T2. In addition, ANG participates in the generation of stress responsive tRFs, which are regulated by its inhibitor, RNH1 [15,23,24,39,40,66]. Interestingly, generation of certain tRFs might be determined by tRNAs with amino acid specificity under different stress conditions [16,29,30,36] or by distinct tissue types [18,20,35,36]. In this tRF biogenesis process, tRNA modifications such as methylation by Dnmt2 and Nsun2 play a key role in the generation and possibly specification of tRF types. One of the suggested functions of tRFs include control of post-transcriptional gene silencing by interactions with AGOs [30,33,35,37,39,45]. In the case of AGO4, interactions with tRFs are expected to be associated with transcriptional gene silencing [12,30]. In addition to controlling transcriptional and post-transcriptional gene expression, tRFs participate in the suppression of gene translation by interactions with ribosomes and the translation initiation complex [56,57,58,59]. Existence of tRFs in human and mouse sera [20,67] as well as in pumpkin phloem sap [68] imply that tRFs might function as a moving signaling component to systematically spread stress responses. In summary, tRFs could modulate stress signaling pathways by induction of gene silencing mechanisms at the transcriptional, post-transcriptional, and translational levels. By immediately reducing gene expression using tRFs, plants can minimize unnecessary cellular responses and maximize survival under stress conditions. Future research that identifies target genes of specific tRF will lead to understanding the mechanism how tRFs downregulate target genes and highlight the function of tRFs in the regulation of stress responses.
